# Investigation on the use of graphene oxide as novel surfactant to stabilize weakly charged graphene nanoplatelets

**DOI:** 10.1186/s11671-015-0882-7

**Published:** 2015-05-08

**Authors:** Salim Newaz Kazi, Ahmad Badarudin, Mohd Nashrul Mohd Zubir, Huang Nay Ming, Misni Misran, Emad Sadeghinezhad, Mohammad Mehrali, Nur Ily Syuhada

**Affiliations:** Department of Mechanical Engineering, Faculty of Engineering, University of Malaya, Jalan Universiti, 50603 Kuala Lumpur, Malaysia; Low Dimensional Materials Research Centre (LDMRC), Department of Physics, Faculty of Science, University of Malaya, Jalan Universiti, 50603 Kuala Lumpur, Malaysia; Department of Chemistry, Faculty of Science, University of Malaya, Jalan Universiti, 50603 Kuala Lumpur, Malaysia; Department of Mechanical Engineering and Advanced Material Research Centre, University of Malaya, Jalan Universiti, 50603 Kuala Lumpur, Malaysia

**Keywords:** Graphene oxide, Graphene nanoplatelets, Weakly charged colloids, Isoelectric point, Hybrid complexes, Electrostatic stabilization

## Abstract

This paper presents a unique synergistic behavior between a graphene oxide (GO) and graphene nanoplatelet (GnP) composite in an aqueous medium. The results showed that GO stabilized GnP colloid near its isoelectric point and prevented rapid agglomeration and sedimentation. It was considered that a rarely encountered charge-dependent electrostatic interaction between the highly charged GO and weakly charged GnP particles kept GnP suspended at its rapid coagulation and phase separation pH. Sedimentation and transmission electron microscope (TEM) micrograph images revealed the evidence of highly stable colloidal mixtures while zeta potential measurement provided semi-quantitative explanation on the mechanism of stabilization. GnP suspension was confirmed via UV-vis spectral data while contact angle measurement elucidated the close resemblance to an aqueous solution indicating the ability of GO to mediate the flocculation prone GnP colloids. About a tenfold increase in viscosity was recorded at a low shear rate in comparison to an individual GO solution due to a strong interaction manifested between participating colloids. An optimum level of mixing ratio between the two constituents was also obtained. These new findings related to an interaction between charge-based graphitic carbon materials would open new avenues for further exploration on the enhancement of both GO and GnP functionalities particularly in mechanical and electrical domains.

## Background

Graphene, a single atomic layer of interconnected carbon atoms in honeycomb configuration, and graphene oxide (GO), which earns its name from the oxidation process of graphite, are known to be the most researched materials at present in academia alongside carbon nanotube [1]. The rapidly growing interest of graphene is predominantly attributed to its remarkable properties which have shown to reach the highest theoretical limit known for the material [2-5]. It is evident from the literature that the most versatile method for producing graphene is via chemical exfoliation of graphite to produce GO followed by subsequent reduction [6-9]. GO is therefore regarded as the most important precursor material to harness the best potentials of graphene. The relatively facile nature in material preparation process and high production scalability have propelled extensive research leading to the expansion of its applications in diverse field such as sensors, energy storage devices, photodetectors, and drug delivery [3,10-12].

GO is known to exist in the form several layers of graphene, and its chemical composition is classified into rich oxidized region where hydrophilic functional groups (i.e., epoxy and hydroxyl at the planar surface and carboxyl, carbonyl, ester, ether, diol, ketone, phenol, quinine, and lactones at the edges) are anchored to sp^3^ carbon atoms as well as pools of un-oxidized graphitic domains which consist of unperturbed hexagonal aromatic chains of sp^2^ bonded carbon atoms [13-15]. The chemical structure of GO renders the material amphifilic in nature similar to that of surfactant and therefore demonstrate high solubility in aqueous-based solvents [14,16-18]. This unique quality inherent within the GO structure enables the material to act as an effective stabilizer to isolate hydrophobic materials such as carbon nanotube and water-insoluble drugs [17-27]. The main advantage of having GO as surfactant to stabilize carbon allotropes lies on its chemical compound which mostly contains conjugated carbon to carbon bonding as well as the two-dimensional configuration which serves to improve material functionalities [28]. Further treatment of GO can be performed in presence of other carbon allotropes which would theoretically enhance the overall composites [19,29-31].

It is generally known that the major limitation towards successful application of carbon nanostructures in vast technological domain is associated to its poor dispersion in both aqueous medium and organic solvents [32-34]. Several approaches have been studied for the production of stable aqueous suspension of carbon nanotubes (CNT) as well as other carbon allotropes for instance [35-40]. Chemical functionalization on nanotube and graphene surfaces via the use highly concentrated acid is a widely adopted technique to increase their solubility [41-44]. However, it is also known that modification via chemical route can disrupt the electronic paths in carbon-based materials due to the opening of the conjugated structure leading to the formation of holes on the surface [45]. This would deteriorate the electrical and other quantum effect properties of the nanotubes as well as other carbon-based structures [46]. To address this issue, some researchers have adopted a non-covalent approach by using surfactant as well as charged and non-charged polymers for dispersing carbon nanotubes along with homogenization and ultrasonication [47-50]. This non-destructive functionalization approach is favorable for attaining defect-free carbon nanotube dispersion since the structural integrity remains unaffected with the grafting of the dispersant molecules although the stabilizing effect is limited [47].

Graphene nanoplatelets (GnP) have recently attained immense attention from research community due to its resemblance to idealized graphene in terms of chemical and morphological structure [51-54]. This material is produced from microwave expansion of acid intercalated graphite which yields a planar structure with average thickness of 1 to 10 nm and varying diameter of 15 to 50 microns [55-57]. The chemical structure of GnP sheets is composed of basal plane layers of aromatic carbon-carbon rings with edges that are prone to oxidation due to the opening of conjugated structure during intercalation and exfoliation processes [51,57,58]. The highly intense microwave radiation up to 2.45 GHz in frequency onto the compactly dense graphite flakes results in thermal expansion as high as 500 times its original volume [55]. The expansion yields a wormlike accordion structure which can be further isolated to produce GnP sheets consisting of few to tens of layers of graphene [59,60].

It was postulated that GnP would demonstrate quality similar to graphene owing to its much pristine basal plane configuration in comparison to GO [57]. GnP has been shown to bring tremendous improvement in diverse applications due to its extremely high optical, electrical, thermal, and mechanical as well as piezoresistive properties [58,61-65]. The defect on GnP edges which mostly consists of unfilled trigonal carbon bonds promotes favorable site for oxidative reaction with ambient gas to yield more hydrophilic structure which comprises various water-based functional groups (i.e., ethers, carboxyls, or hydroxyls) [51,58]. Thus, it can be said that the solubility of GnP is size-dependent since more functional groups exist in larger size sheets which contain more structural defects sites [66]. The presence of these functional groups also paves avenues for further covalent and non-covalent functionalization to enhance its solubility [52,67].

It has been proven that the close resemblance of GnP sheets to graphene mono-structure implies that the former may closely exhibit enhanced thermal and electrical conductivity demonstrated in graphene [4,65,68,69]. Due to its unique planar morphology, GnP may offer higher thermal contact area to minimize the thermal resistance especially at low loading levels, enabling much higher thermal conductivity to be attained in comparison to buckeytube carbon-based structures [65,70]. Researchers have used GnP as a filler material for producing composite structures with promising thermal and mechanical potentials [55,56,71-76]. Further, recently, Do and coworkers [77] have proposed GnP as a low-cost but effective base material comparable to carbon nanotube and carbon black to further enhance fuel cell catalytic property as well as promote high-level graphitization and resistance to thermal oxidation.

It is evident that the stability of carbon-based materials in an aqueous medium strongly relies on the concentration of hydrophilic functional groups which interact via hydrogen bonding and columbic repulsive force [14,78]. The degree of particle stability is commonly interpreted in terms of morphological and quantitative perspectives [9,13,14]. For GnP which consists of hydrophilic groups strategically localized at the particle edges, its suspension stability is highly dependent on particle size, and unlike GO, the much pristine basal structure consisting of aromatic carbon rings promotes high tendency for hydrophobic interaction between particles which increases the rate of agglomeration and sedimentation [73,79].

Much effort has been devoted to achieve a stable suspension of graphite nanoplatelets with various surfactants and polyelectrolytes (PEs) [52]. A common quantitative approach for classification of stable colloid is based on zeta potential measurement which provides information on the level of electrostatic repulsion between particle surfaces. It is evident from the literature that a zeta potential value of ±35 mV would normally signify a stable colloidal suspension [52,80] and magnitude of higher than 60 mV would indicate excellent stability [80,81]. However, as mentioned above, the unique distribution of oxygen-based functional groups around GnP peripheral edges may result in misleading interpretation of zeta potential measurement as highlighted by Lu et al [52]. The actual particle dispersion may take into account the strong hydrophobic interaction at the particle basal plane on top of the electrostatic repulsive nature of its edges. Thus, further modification on the particle solubility is imperative in order to extend its potential as well as harnessing its remarkable properties for diverse applications.

In the present research, GO was used as novel dispersant to stabilize GnP particles in order to improve its processability. It is believed that combination of these elements will ensure all carbon colloidal mixtures without participation of organic surfactants which effectively address the surfactant removal issues. It is postulated that π-π interaction between the aromatic structure of both constituents may hold the key for ensuring successful stabilization of GnP as demonstrated by earlier studies on carbon nanotube dispersion using GO [18,19,21-23,25,31,82,83]. On top of that, the hydrophilic functional group anchored at the peripheral edge of GnP would theoretically interact with abundant oxidized sites of GO at both the basal plane and the edges to improve the overall stability of GnP colloids.

Further, due to the high stability of GO-based aqueous solution at a wide range of pH which is evident from the zeta potential value [17], special attention will be directed on exploring its role towards enhancing the stability of GnP suspension near its point of zero charge (PZC) where particle aggregation and sedimentation dominate. This is based on the previous discoveries on the successful application of highly charged particles to stabilize extremely weak colloidal system via ‘haloing effect’ [84,85]. It is postulated that a virtually similar interaction may manifest for the present colloidal system which consist of a mixture of highly stable GO and pH-sensitive GnP which is known to rapidly flocculate at lower pH [63]. It is also anticipated that the incorporation of GnP on GO via the above interaction will serve to improve the overall performance of the composite structure by specifically targeting the defect site of GO as platform to complete the interaction. Thus GnP can also act as colloidal patch to repair defects in GO structure which occur during its synthesis as demonstrated by previous researches [18,21,29].

## Methodology

### Material

The chemicals used for sample preparation are given as follows: Sulfuric acid (H_2_SO_4_, 98%), phosphoric acid (H_3_PO_4_, 85%), potassium permanganate (KMnO4, 99.9%), and hydrogen peroxide (H_2_O_2_, 30%) were obtained from Merck (Darmstadt, Germany). Hydrogen chloride (HCl, 37%) and ssodium hydroxide (NaOH) was purchased from Sigma-Aldrich (St. Louis, MO, USA). Expendable graphite flakes (grade 3061) were received from Asbury Graphite Mills Inc (St. Asbury, NJ, USA). Highly purified de-ionized water from Thermo Scientific™ Barnstead™ NanoPure™ system (Thermo Scientific, Waltham, MA, USA) with resistance of 18 MΩ.cm was used for the preparation of stock solutions. Graphite nanoplatelet powder (grade C) was purchase from XG Sciences, Inc. (Lansing, MI, USA) with average specific area of 500 m^2^/g.

### Preparation and characterization techniques

The GO preparation followed similar procedure as described by Marcano et al. [86] and further simplified by Huang et al [87]. In a typical procedure, H_2_SO_4_ and H_3_PO_4_ with 320: 80 ml ratio along with 3 g of graphite were mixed in a 2-l beaker under stirring mode. Eighteen grams of KMnO_4_ was subsequently poured into the mixture and left for 3 days to allow complete oxidation. At the end of the oxidation process, about 27 ml H_2_O_2_ was added into the solution to terminate the oxidation. The solution was subsequently washed with 1 M HCL and deionized water (DI) water under centrifugal force up to 11,500 g using high-speed refrigerated centrifuge unit from HITACHI (model CR21Fiii; Hitachi, Tokyo, Japan) until reaching appropriate pH. The centrifugal process allowed the highly oxidized graphite to dissociate to its individual GO sheets consisting of several atomic layers. About 1 ml of the highly concentrated hydrogel product was finally weighed and dried at 60°C for 3 days to determine the actual concentration.

For preparing GO-GnP sample, GnP was first mixed in a 60-ml sampling bottle of DI water solution and underwent sonication for 2 min at 50% amplitude using ultrasonic processor (Sonics Vibra-Cell, VCX 750, Sonics & Materials, Inc., Newtown, CT, USA) with a 13-mm probe. Next, an appropriate amount of GO corresponding to the desired weight percentage was taken from the stock solution and mixed with the GnP solution. Vortex mixer was subsequently used for dispersing the mixture to preserve the GO size. The pH was later adjusted using 1 M HCL and NaOH to reach the desired value while keeping the solution in stirring mode to promote homogenized chemical reaction. The sample was finally undergone series of testing and characterization processes to further observe, analyze, and verify the hypotheses.

### Sample characterization

Characterization of the sample was performed using the following analytical devices: The Raman spectral data were reduced via Renishaw inVia Raman microscope system (Renishaw, Gloucestershire, UK) equipped with a 514-nm laser beam. Ultraviolet-visible spectral data were extracted from Varian carry^@^ 50 UV-vis spectrophotometer from Agilent Technology (Santa Clara, CA, USA) with dual beam configuration and Xenon flash lamp as light source. The Fourier transform infrared (FT-IR) spectral data were obtained from Perkin-Elmer-FT-IR spectrum 400 (PerkinElmer, Waltham, MA, USA). The crystalline phase was determined using a Phillips X-ray diffractometer (XRD) (Phillips, Amsterdam, Netherlands) employing a scanning rate of 0.033°s^−1^ in a 2*θ* range from 5° to 80° with Cu Kα radiation (*λ* = 1.5418 Ǻ). The physical structure of the particle was classified using TEM LEO LIBRA-120 (Carl Zeiss, Oberkochen, Germany). Hydrodynamic size and zeta potentials of the particles were measured by Zetasizer Nano ZS (Malvern Instruments Ltd, Malvern, UK) using 4 mW He-Ne laser operating at a wavelength of 633 nm with detection angles of 173° and 13° for size and zeta potential measurements, respectively. Rheological examination was conducted using Anton Paar Rheometer (model Physica MCR 301, Anton Paar GmbH, Graz, Austria) equipped with double gap concentric tools.

## Results and discussions

### Characterization of GO, GnP, and GO-GnP hybrid mixture

**Figure 1:**
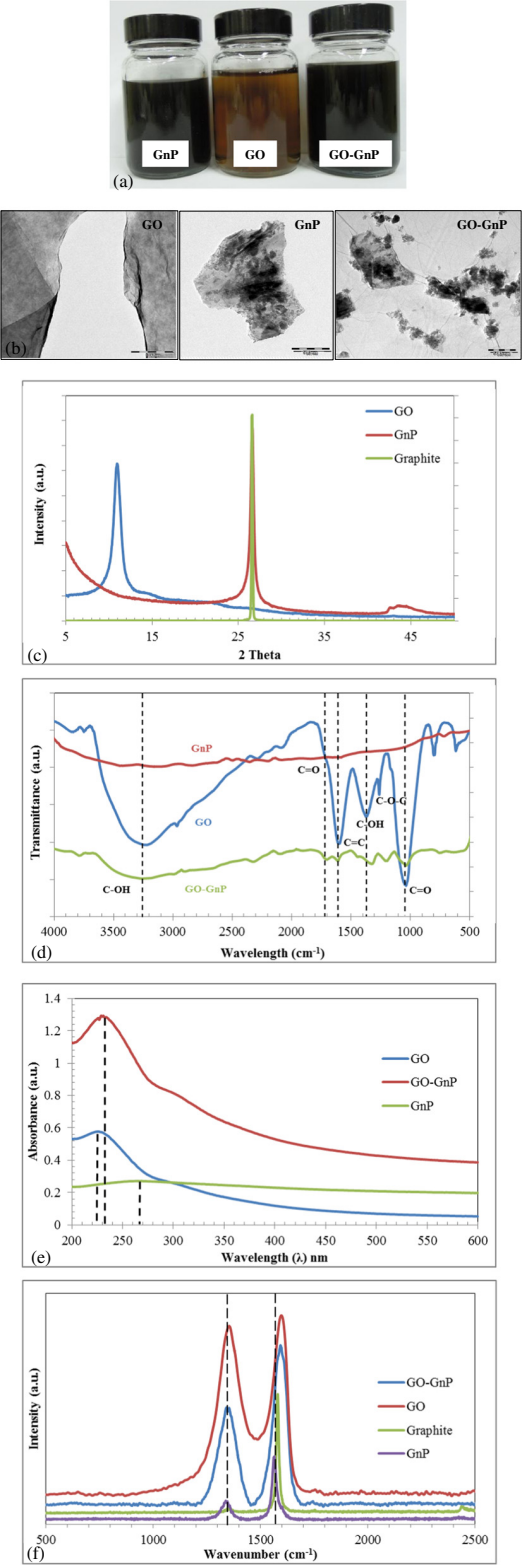
Typical characterization route for GO, GnP, and its hybrid mixture (GO-GnP). **(a)** Sedimentation image, **(b)** TEM micrograph, **(c)** XRD, **(d)** FT-IR spectra, **(e)** UV-vis spectra, and **(f)** Raman spectra.

Figure 1a,b,c,d,e,f highlights on the conventional route for identification of GO, GnP, and their hybrid mixture. The colloidal picture provides a fundamental glance on the color and dispersion level of each element in aqueous solution as provided in Figure 1a. The brownish color of GO signifies a successful oxidation process that originates from electronic transition of its molecular orbitals [9,87,88]. On the other hand, a much darker solution containing GnP particles manifests which suggests the unperturbed conjugated structure of its basal plane reminiscence to graphite [2,89-91].

The TEM micrograph from Figure 1b clearly shows the morphological structures of GO which consist of a flake-like formation with wrinkles. GnP particle on the other hand appears to be irregular in shape mainly due to the route of its production which involves extreme thermal expansion and high energy sheet isolation processes [55]. Further, some fragments of much smaller GnP particles were seen entrapped on the basal plane which may occur due to size reduction phase involving pulverization process. For GO-GnP hybrid configuration, it was shown that GnP particles were mostly anchored onto GO sheet which was mostly attributed to the electrostatic and hydrophobic interaction between the particles.

As given in Figure 1c, XRD results show different diffraction peaks between GO, GnP, and graphite (i.e., 10.9° for GO, and between 26.4° and 26.5° for both GnP and graphite flakes) [92-94]. This is largely due to the change in interlayer spacing for GO to that of graphite and GnP flakes (i.e., 0.87 nm and 0.34 nm, respectively). The distance between consecutive sheet layers was increased for GO due to the presence of hydrophilic functional groups at the GO basal plane originated from the chemical oxidation.

FT-IR measurements on GO as highlighted in Figure 1d verify the existence of various water-based functional groups formed during the oxidation process as well as the preservation of conjugated aromatic ring at the basal plane [9,13,95]. This will render the colloid highly soluble in aqueous-based solution [14]. On the other hand, virtually no significant oxygen-based functional group peak exists to classify GnP hydrophilic nature suggesting a highly pristine graphitic structure similar to the characteristic of CNT [30,43]. Interestingly, the addition of GnP on GO resulted in the appearance of several prominent peak of hydrophilic-based functional groups on the GnP spectral background indicating the enhancement in the solubility of the GnP colloids.

UV-visible spectral measurement of GO and GnP shows that different peaks manifest for each of the constituents as depicted in Figure 1e. The peak at 225 nm for GO was due to the π → π* transition of the C = C bonding, which is similar to the reported value in the literatures [19]. Meanwhile the shoulder peak around 300 nm was attributed to *n* → π* transition of the carbonyl groups [87]. The much lower maximum peak wavelength of the present GO signifies the increase in oxidation sites with higher distribution of functional groups at the basal plane [17,87,96]. For GnP, the peak around 269 nm was observed which denotes the C = C bonding of the aromatic structure along its basal plane. These results mostly concur with carbon-based materials in the literature [63,96,97]. The plot for GO-GnP hybrid mixture elucidates the red-shifting of maximum peak wavelength from 225 nm to 231 nm. This is predominantly due the effect of GnP anchoring on GO basal structure which was also observed previously in GO-CNT hybrid mixture [26,30]. Further, the addition of GnP into GO-based solution increases the intensity of light absorbance which translates into a high level of stability of the system.

Figure 1f shows comparative assessment of Raman spectral measurements between GO, GO-GnP hybrid mixture, and graphite. The results indicate that graphite is distinctively characterized by a prominent G-peak at around 1,580 cm^−1^ which is a common signature of the first-order scattering of E2g phonon from sp^2^ carbon graphitic lattice [98,99]. On the other hand, the Raman spectroscopy measurement for GO unveils a combination of nearly equal composition of G and D peaks. The D peak is commonly interpreted as structural defect on the basal plane as well as signifying the intensity of sp^3^ carbon molecular structures where the hydrophilic functional groups are appended [83,100]. The high intensity of D peak vindicates the previous UV-vis spectral analysis on the decrease in π-conjugated structure within the GO basal plane [17]. This feature may exhibit intrinsic benefits particularly in further processing of GO such as functionalization and composite material fabrication and could also potentially act as colloidal stabilizer [17,19,101].

It is also evident that both D and G bands of GO-GnP were blue-shifted to a much lower value (i.e., D-1,348 cm^−1^, E-1,593 cm^−1^) from GO (i.e., D-1,354 cm^−1^,E-1,598 cm^−1^) to match the pristine graphitic structure inherent within GnP material (i.e., D-1,340 cm^−1^, E-1,564 cm^−1^). Further, the peak intensity ratio (i.e., I_D_/I_G_) value of the hybrid mixture drops almost double from GO which probably suggests that most of the sp^3^ bonds that connect the carbon atom to the water-based functional groups are overlaid by GnP. This result is almost similar to the study conducted by Cheng et al. [102] on the evolution of peak intensity ratio of repaired GO which shows a decreasing trend signifying the rise of sp^2^ carbon intensities within the GO sites [99,103]. The above ratio is generally understood as representing the degree of crystallization and the alignment of the graphitic planes of the carbon materials [99]. The unique mobilization of GnP also serves to repair the defect structure within GO where this sp^3^ bond mostly manifests. These results strongly suggest the presence of GnP colloid in GO matrix.

### Investigation of GO-GnP colloidal stability via imaginary technique

**Figure 2:**
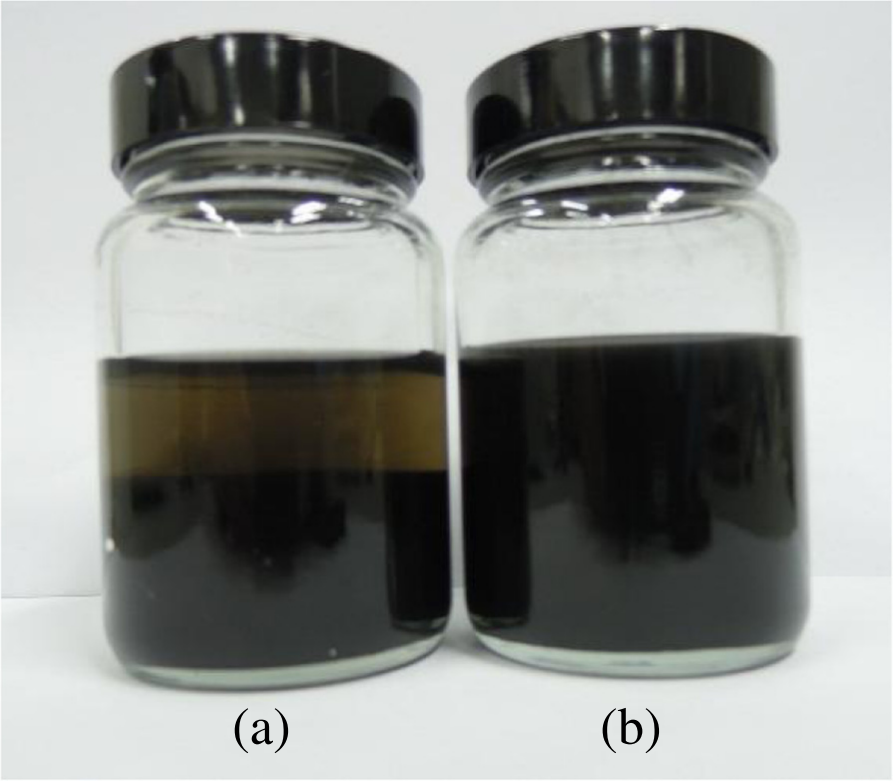
Sedimentation image of GO stabilized GnP. Sedimentation image of GO stabilized GnP at **(a)** pH = 8 and **(b)** pH = 3.2 taken after 2 months.

**Figure 3:**
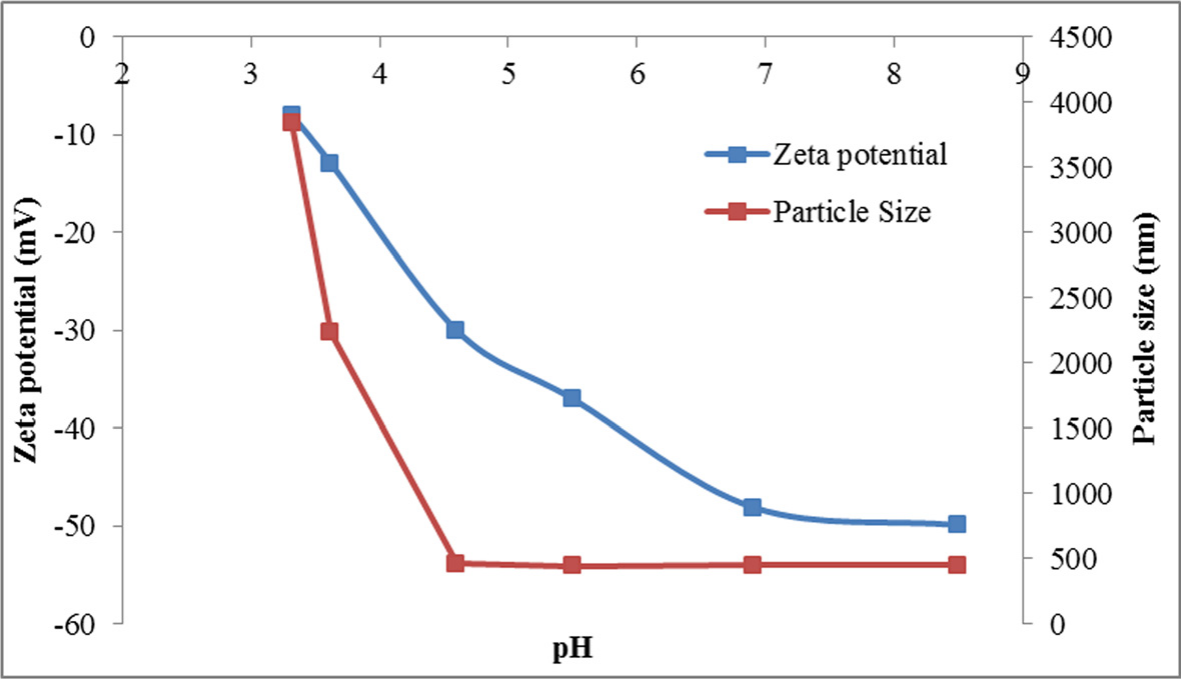
Plots of particle size and zeta potential of GnP with respect to pH.

**Figure 4:**
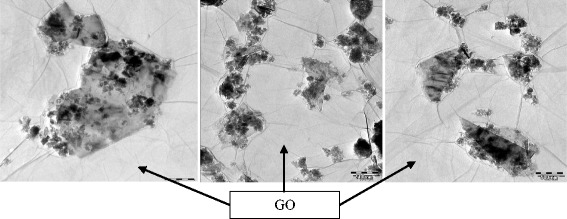
TEM micrograph of GO-GnP hybrid structure at pH 3.2.

Figures 2, 3 and 4 show the sedimentation image of the GO stabilized GnP at two extreme pH, the zeta potential measurement of individual GO and GnP colloids as well as the TEM micrograph of the morphological architecture of the hybrid colloidal system. These results strengthen the fact that GO can act as efficient stabilizer even at the pH of flocculation for GnP. This interesting finding warrant further investigation onto the mechanisms involved in the interactions between particle constituents within the colloidal mixture. It is generally known that charged particles are prone to rapid aggregation, flocculation, and sedimentation as the zeta potential reaches the unstable region [96]. This can be illustrated by Figure 3 that shows the rapid increase in GnP particle hydrodynamic size as the pH shifts closer to the isoelectric point. However, the addition of GO into this unstable colloidal system exceptionally improves the dispersion level of GnP which was shown by the sedimentation image. Moreover and quite surprisingly, the GnP remained in a stable form even after 2 months.

In contrast, a similar sample prepared at pH 8 which is known to be the stable region for both GO and GnP colloids exhibits a sedimentation feature which is evident by the presence of two separate phases. As highlighted in Figure 4, a TEM micrograph shows that GnP remains intact and becomes entrapped on the GO basal plane and between its edges. It is believed that the GnP particles mostly fill up the void that may constitute the defect which is the common morphological character of GO [13]. The size of the GnP is mostly preserved and remains discrete similar to its individual structure. The results strongly suggest that GO can enhance the colloidal stability of GnP which may pave avenues for much facile and scalable processing of carbon-based materials. For instance, a much enhanced lateral and out of plane electrical, thermal, and mechanical properties of GO-GnP thin film composites are expected upon its reduction process which may pave ways for producing highly efficient, compact, and lightweight physical process instruments such as heat exchange and phase change devices [104].

**Figure 5:**
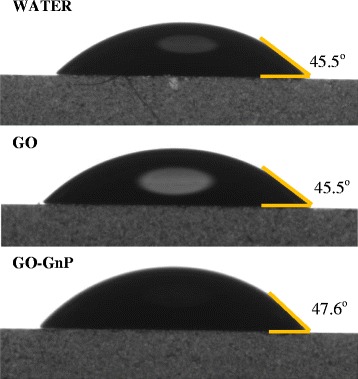
Contact angle measurement of different colloidal elements. GO-GnP hybrid mixture was prepared at pH 3.2.

**Figure 6:**
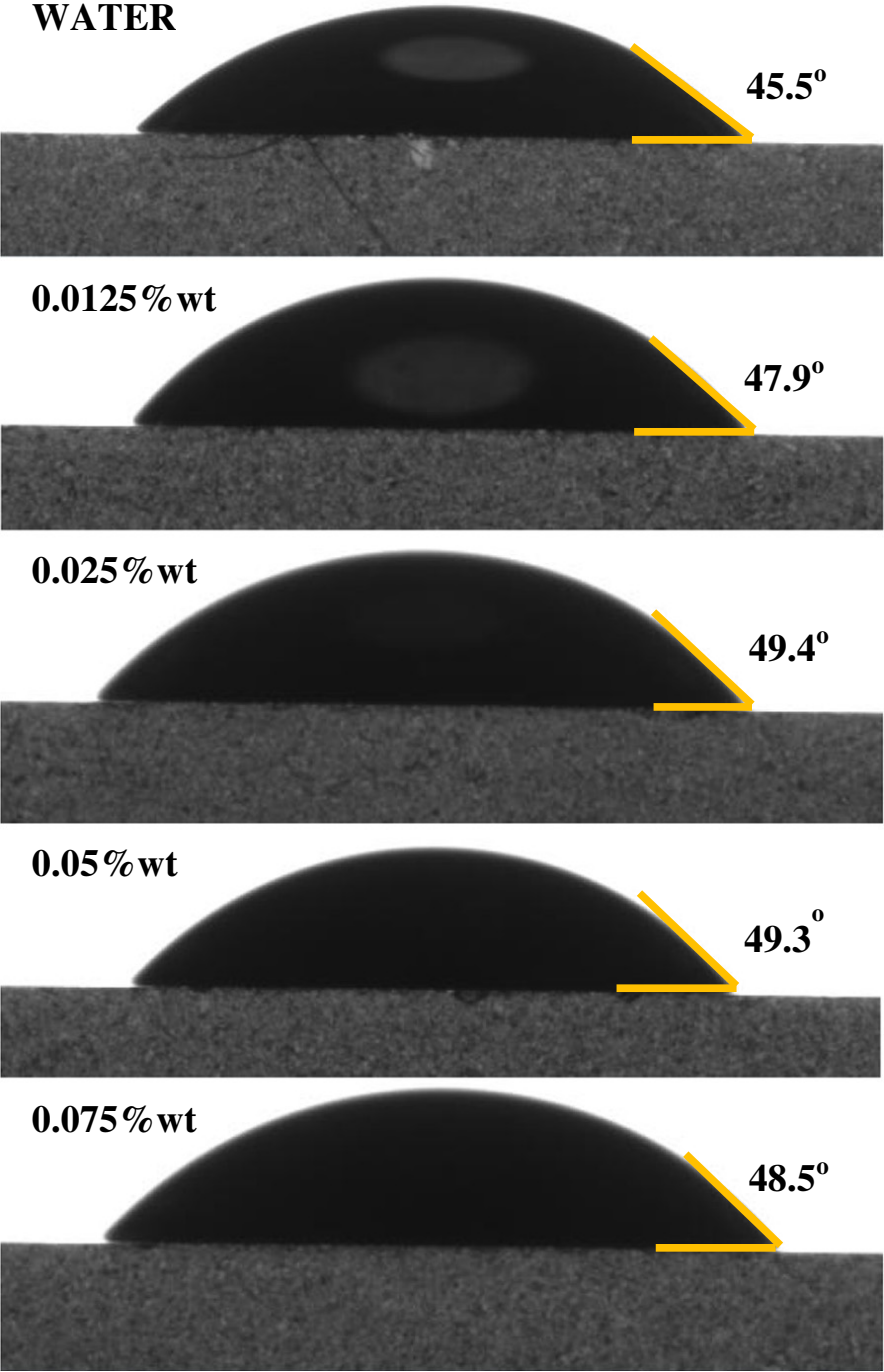
Contact angle measurement of different amounts of GnP elements. Contact angle measurement of different amounts of GnP elements in GO-GnP colloidal mixture at pH 3.2. GO concentration was fixed at 0.025 wt%.

Figures 5 and 6 show the comparison of contact angle measurement between GO-GnP, GO, as well as water to give an insight on the effect of pH and particle loading onto the wettability of the colloidal system. The results from Figure 5 show that adding GnP and changing the pH close to its isoelectric point causes a relatively small increase in contact angle measurement in comparison to the corresponding GO and water value. This indicates that GO successfully prevents GnP from experiencing rapid flocculation due to the intensifying attractive van der Waals forces within the colloidal system leading to the expansion of contact angle value. As evident in Figure 6, the results for different GnP concentration show that the wettability is reduced in accordance to the increase in GnP concentration which signifies the increase in hydrophobicity of the colloidal system. Nevertheless, these increments are rather small to enable complete alteration of the contact angle to minimize the surface tension. The affinity towards glass plate was still preserved which was mostly attributed to a well-dispersed system.

### Investigation of GO-GnP colloidal stability via electrophoresis technique

**Figure 7:**
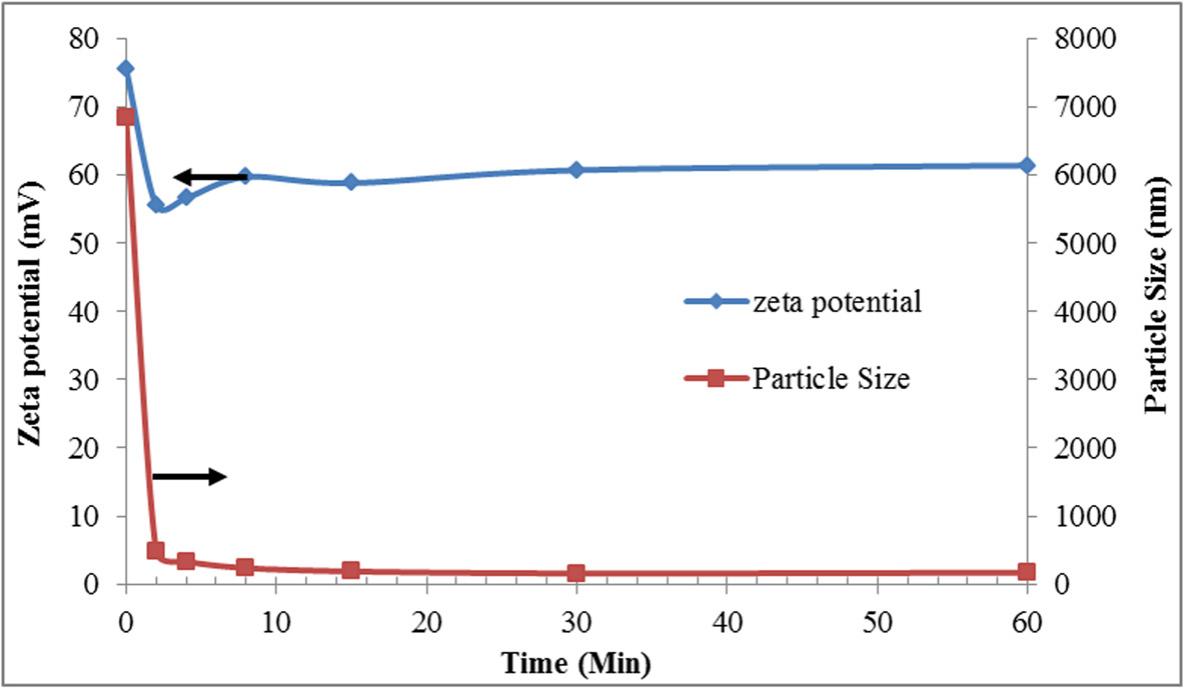
Particle size and zeta potential value of GO with varying sonication time.

Figure 7 shows the size and zeta potential measurements of GO at different sonication times and pH. These results explain the reason behind the excellent stability of GO which exhibits high zeta potential value. It was known that zeta potential of more than 60 mV in magnitude indicates high stability of colloidal solution [81]. This is due to strong repulsive electrostatic forces between adjacent particles originating from high-density electrical charges formed during deprotonation of various functional groups. The figure shows a dramatic drop of zeta potential value after just 2 min of sonication treatment using a probe sonicator. This shows that different sizes of GO particles demonstrate distinctive characteristic of colloidal stability. The as prepared GO contains abundance of oxygen functional groups (hydroxyl, carboxyl, and carbonyl) which render high density of electrical charge per unit area. The collapse of GO structures during sonication process into much smaller fragments may change the charge density surrounding the particles which led to abrupt drops in zeta potential.

**Figure 8:**
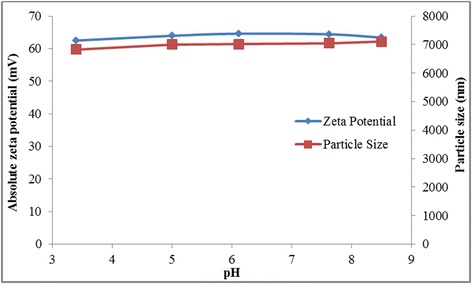
Absolute zeta potential and particle size value of GO at different pH.

Further sonication under similar power intensity may have little effect of the GO overall stability since the particles may have been broken into much smaller pieces during the initial period of sonication which was evident from the plot of particle size. However, the zeta potential value is still above 50 mV which falls within the high stability region, and the particles was observed to remain suspended for months. Further, Figure 8 shows that the GO colloid produced demonstrates high robustness against changes in pH. This is also attributed to a large oxidized area within GO produced using the present approach as highlighted by Marcano et al. [86] and was later proven from the peak wavelength absorbance that was blue-shifted to 225 nm indicating an increase in sp^3^ domain [17,87].

**Figure 9:**
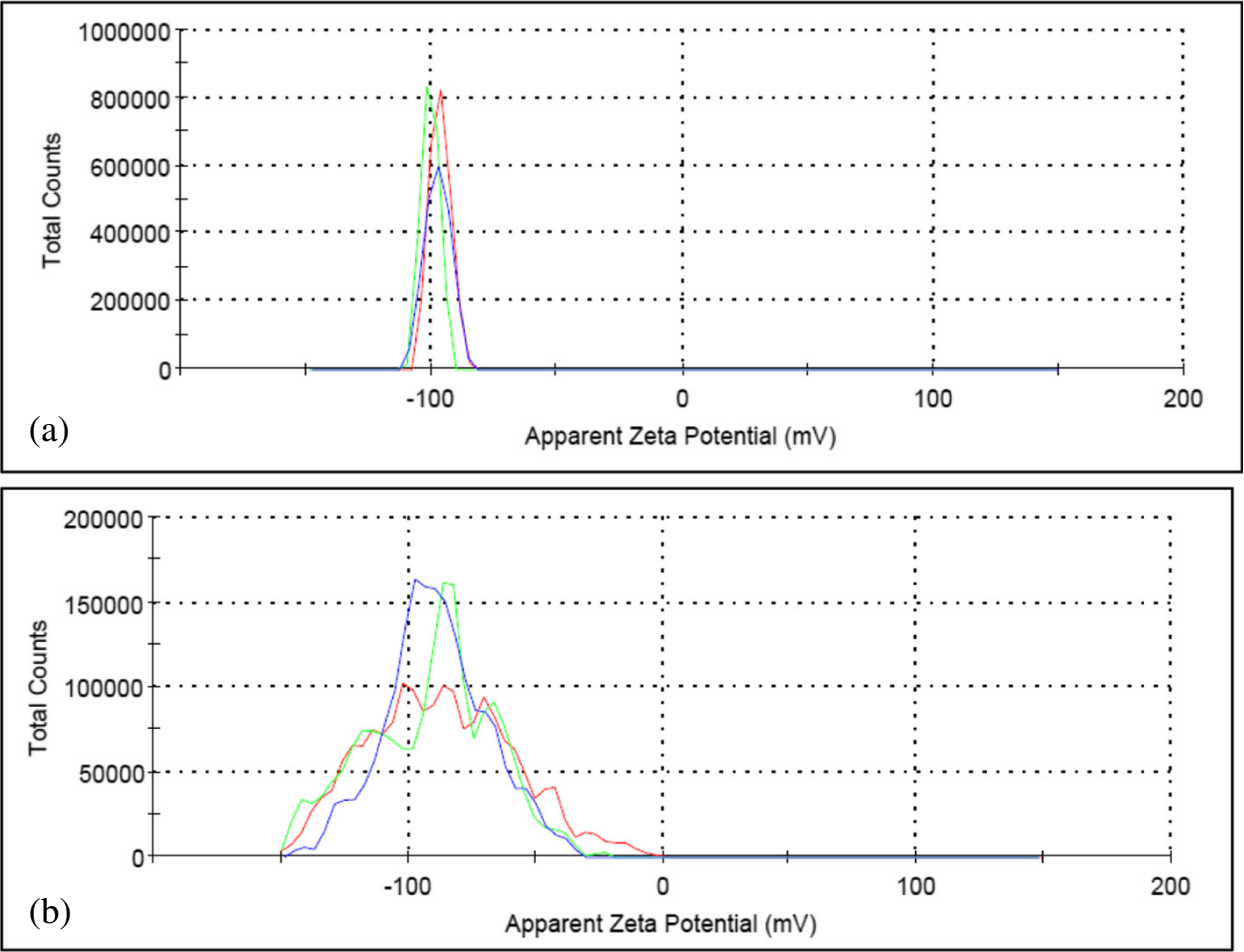
Zeta potential distribution curve for GO-GnP at **(a)** pH 3.2 and **(b)** pH 8.

Figure 9 shows the zeta potential statistical curve for GO-GnP hybrid mixture at two different pH. It is evident that zeta potential measurement of the sample where the pH was adjusted to pH 3.2 shows much lower deviation in comparison to the sample prepared at pH 8 by which the statistical distribution was broad and relatively poor. Further, the magnitude of zeta potential for the composite mixture at the lower end of pH is much higher in comparison to individual constituent (i.e., GO = −70 mV and GnP = −10 mV). This interesting observation shed light onto the unique interaction between particles within the colloidal matrix. For solution in an extremely basic condition, both GnP and GO are in a stable form which is evident by the zeta potential value (i.e., GO = 60 mV and GnP = 45 mV). Thus both particles are mutually repulsive, and since the zeta potential is measured based on the electrophoretic mobility of the particles [105], the sensor translates the frequency shift in the light scattering components into zeta potential value for individual element which results in a much broader distribution. In addition, GnP is generally known to have much preserved aromatic structure on its basal plane which is hydrophobic in nature. In this context, the colloidal stability is only provided by its parametric edges which is more hydrophilic due to existence of water-based functional groups [51,52]. Therefore, the competing repulsive electrostatic and attractive van der Waals forces between GnP particles and GO sheet result in large fluctuation of measured zeta potential.

On the other hand, at first glance, it was thought that the GnP in GO-based solution would rapidly flocculate and settle down as the pH was lowered down to 3.2 in magnitude. However, based on the zeta potential results, it was postulated that the particles strongly interact with GO via columbic force and remaining close to the hydrophilic site of GO without mutual contact. This phenomena is similar to the one discovered by Tohver et al. [85] which shed light on the novel stabilizing mechanism of asymmetrically charged colloidal system. The abnormally high zeta potential value for the hybrid system was also reported previously by Shu Xi et al. [106] and Herman et al. [107] which was probably caused by the small separation distance between the interacting particles, closed to the Dybe length of each constituent. Herman et al. [107] attributed this anomaly to the limitation on the electrophoretic measurement that is highly dependent on particle sphericity.

It is evident that the change in zeta potential value strongly signifies the existence of a unique interaction between the particles. Further, while previous studies focus on understanding the stabilizing effect of low charged microparticles with highly charged nanoparticle [108-112], the present study embarked on the opposite configuration wherein the GnP particles become highly attracted to a much larger GO sheet, but the repulsive nature of both elements prevents their permanent contact within the bimodal system. This result coupled with the imagery evidences may strongly suggest that the hybrid composite mixture exhibits the prevalent characteristic of highly stable colloid which is mutually attractive in a long range but sufficiently repulsive as both particles are drawn closer within the Dybe length zone [113].

**Figure 10:**
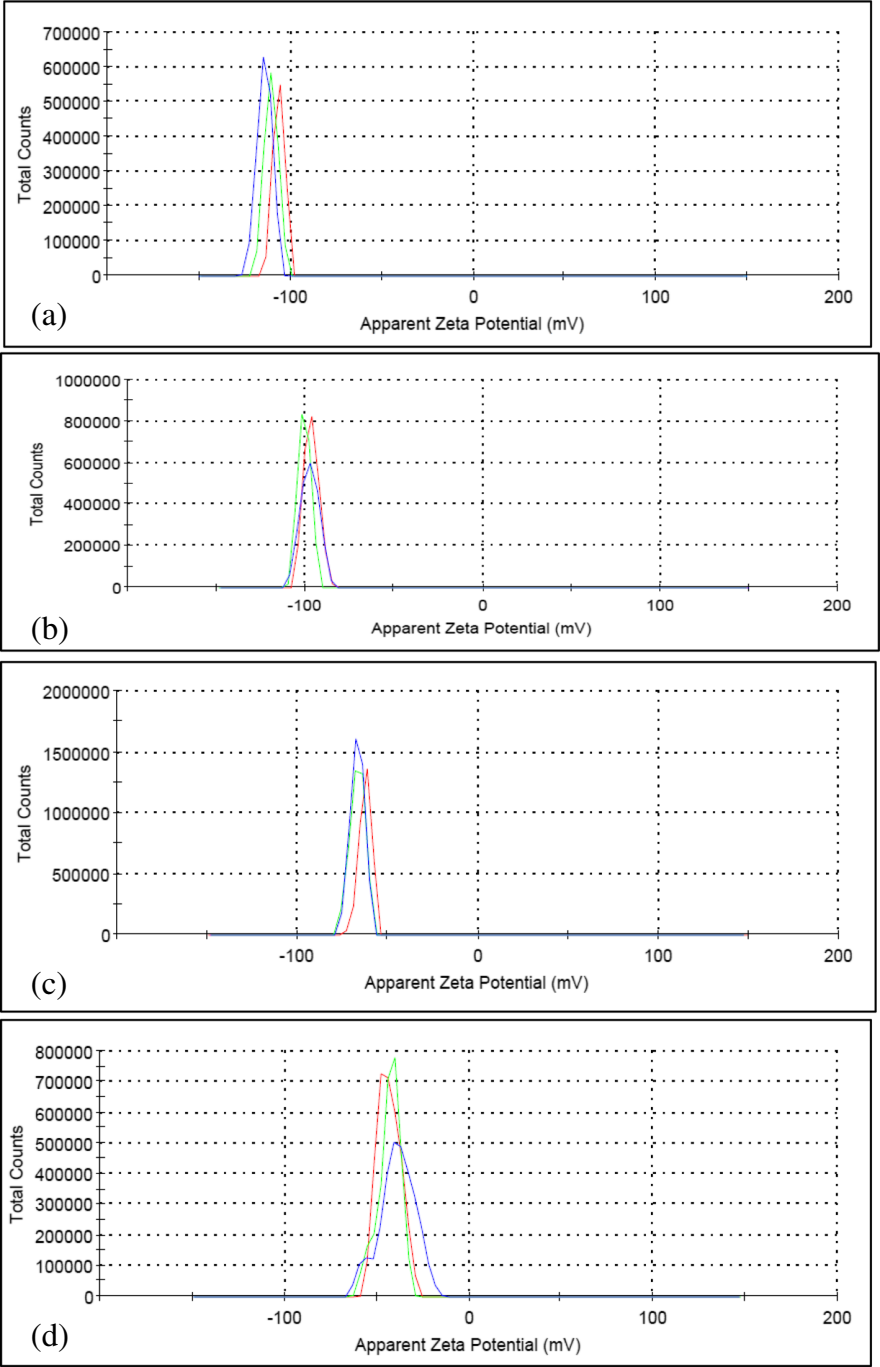
Zeta potential distribution curve for GO-GnP at different GnP concentrations. **(a)** 0.0125, **(b)** 0.025, **(c)** 0.05, and **(d)** 0.1 wt%. GO concentration was fixed at 0.025 wt% and the pH was kept at 3.2.

Figure 10 shows the zeta potential measurement for series of GO-GnP colloidal mixtures consisting of different amounts of GnP at specific GO concentration. The results show that the zeta potential value dropped in accordance to the increasing GnP concentration. This is highly expected since the site where GnP particles anchor will become saturated, and additional particles will remain in suspension that causes the overall stability to drop. This loading-sensitive stabilization behavior was also documented previously that outlines the effect of particle concentration and size ratio between high- and low-charged particles on altering the window of stability of colloidal mixtures [84,85,112,114]. However, it is evident from the results that the stability of GnP in the colloidal system remains high even at four times the concentration of GO which justifies the underlying importance of GO to enhance the stability of different colloids.

### Investigation of GO-GnP colloidal stability via rheological analysis

**Figure 11:**
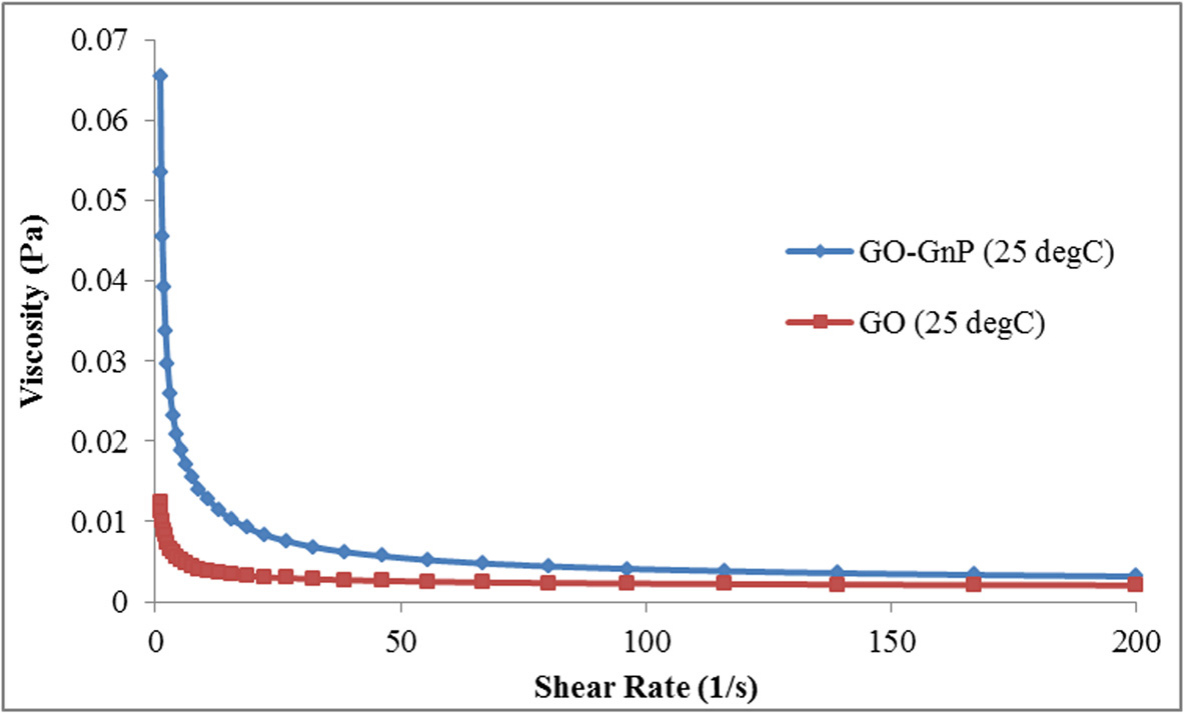
Plot of viscosity against shear rate for GO and GO-GnP hybrid at 1:1 ratio. Both samples were prepared at 0.05 wt%.

**Figure 12:**
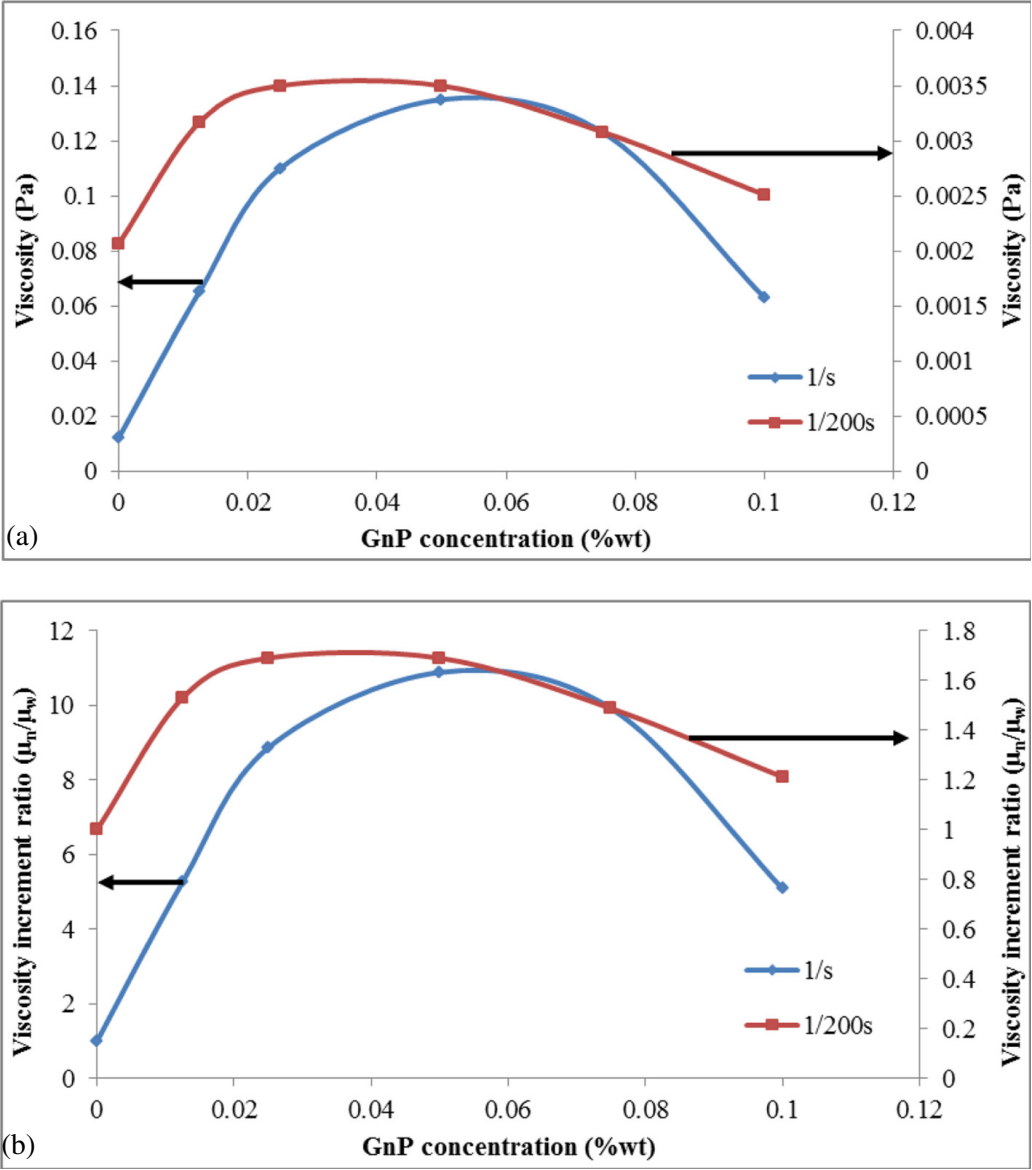
Plot of **(a)** viscosity and **(b)** viscosity increment ratio with respect to GO at varying GnP concentrations. GO concentration was fixed at 0.025 wt%.

Figures 11 and 12 provide a closer look onto the rheological perspective of the hybrid mixture. It was observed that the addition of GO on the solution containing GnP colloid causes a significant change in viscosity magnitude at a specific shear rate as evident from Figure 11. The viscosity of the mixture increases more than eight times the viscosity of GO alone at a shear rate of 1/s. As the shear rate increases, the viscosity drops sharply to match the reference GO value, and at 1/200 s shear rate, the viscosity increment drops to 1.6 times the value of GO. This interesting finding may explain the increase in stability of GnP. The interconnected unstructured network of GO and GnP particles tremendously increases the flow resistance at a low shear rate while the increase in shearing force at higher shear rate causes the highly mobile GnP to hover on the GO sheet. This paves way for rapid realignment of the particle to reduce particle-particle and particle-fluid frictions and facilitate a viscous motion.

Figure 12a,b elaborates on the effect of increasing the amount of GnP on the overall viscosity measurement. It shows that increasing the GnP concentration results in the rise of viscosity at both low and high shear rates. The highest increment was recorded at two times the concentration of GO. This implies that the interaction between GO and GnP is the highest at this combination which also suggests that much of the hydrophilic sites on GO are occupied by GnP. Previous studies have also highlighted an optimum concentration range of colloidal mixtures to yield a stable hybrid system [84,115,116]. As the GnP concentration increases beyond this benchmark, the viscosity rapidly drops which may indicate the saturation of GnP on GO sheet and the remaining particles will strongly flocculate and form much bigger structures. This will alter the particle-particle and particle-fluid interaction within the colloidal mixture that would lead to the modification of the momentum transfer and eventual lowering of viscosity.

### Investigation of GO-GnP colloidal stability via particle absorbance measurement

**Figure 13:**
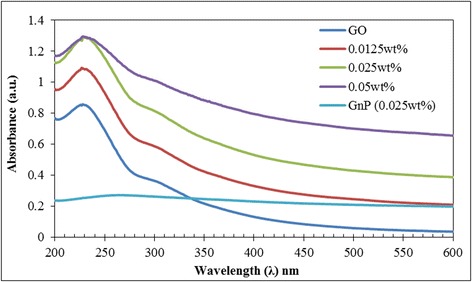
Plot of absorbance versus wavelength for GO-GnP at different loadings of GnP. GO concentration was fixed at 0.025 wt%.

**Figure 14:**
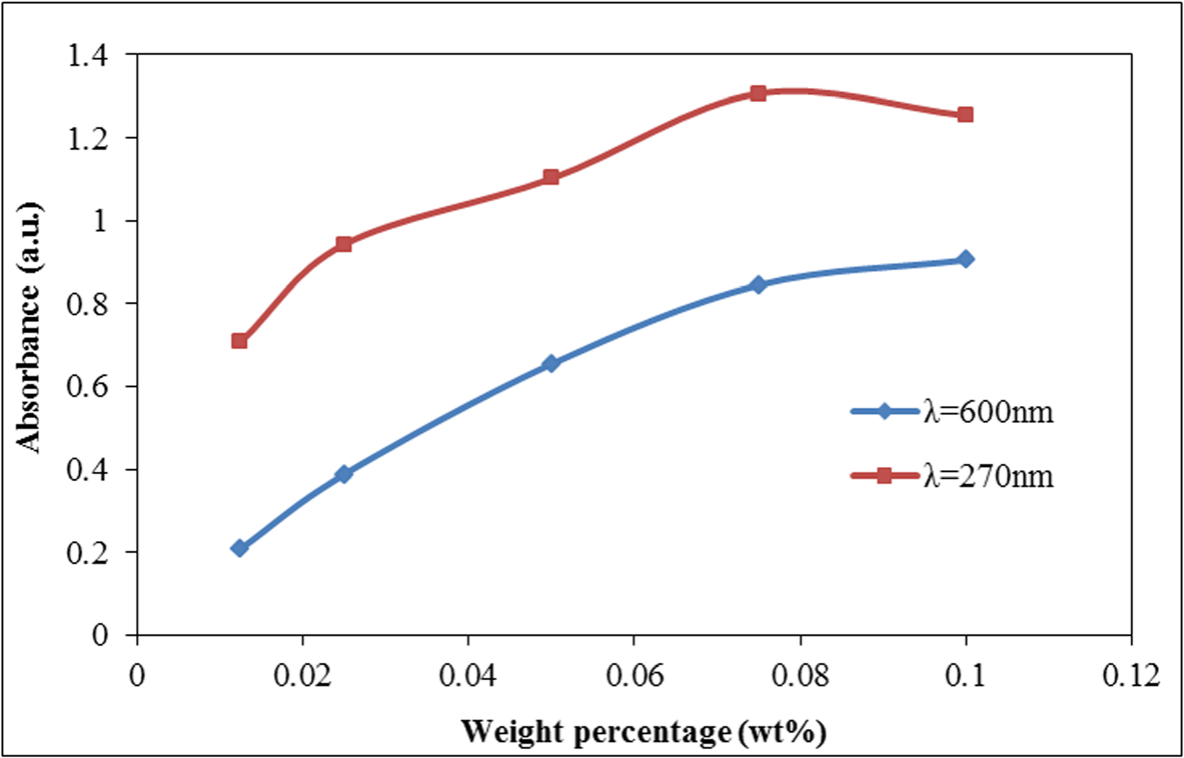
Plot of absorbance at different concentrations of GnP at a specific wavelength. GO concentration was fixed at 0.025 wt%.

Figures 13 and 14 present the plot of light absorbance measurement on the colloidal solution at a specific range of wavelength which also gives an insight on the level of stability of the suspended particles. It was elucidated from Figure 13 that the addition of GnP into a GO-based solution increases the intensity of light absorbance which reflects a significant improvement on the stability of the system. Further, the rate of absorbency increment is much higher at low particle loading and gradually subsides with rising GnP concentration as shown in Figure 14. These findings are also in congruent to the previous approach on measuring particle light absorbance to determine the stability of weakly charged colloids in highly charged nanoparticles [107]. The results also vindicate the existence of optimum stabilizing window for the colloidal mixture to avoid flocculation and phase separation [84].

## Conclusion

The present report highlighted for the first time the unique synergistic interaction between two different graphitic carbon structures, each having different charge strengths that enable a highly stable colloidal mixture to be accomplished. The results showed that GO, owing to its unique chemical and morphological structures, can be exploited as dispersant to effectively maintain GnP colloidal stability near its negligibly charged state (i.e., lowest charge density) where it was generally known to experience rapid aggregation due to extremely low repulsive forces. Series of measurements suggested that the interaction between the two colloids is mutually attractive in the long range but sufficiently repulsive in nature at a limited distance between the particles to prevent irreversible contact. Further, it was believed that the hovering of GnP particles on the planar and edges of charge-dominating GO structures prevents further contact between GnP particles. The imagery, electrophoresis, and rheological measurement techniques consolidate the existence of different physics to describe the stabilizing effect within the binary system. It is anticipated that this interesting behavior may provide favorable condition to further enhance the material property and expand the application frontier in science and technology.

## Abbreviations

DI, deionized water; FT-IR, Fourier transform infrared; GnP, graphene nanoplatelets; GO, graphene oxide; T, temperature, K; TEM, transmission electron microscope; UV, ultraviolet; XRD, X-ray diffraction.

## Greek

wt%, weight percentage; μ, viscosity, Pa

## Subscripts

w, water; n, nanocolloids
